# Endoplasmic reticulum stress in hepatic steatosis and inflammatory bowel diseases

**DOI:** 10.3389/fgene.2014.00242

**Published:** 2014-07-25

**Authors:** Beichu Guo, Zihai Li

**Affiliations:** ^1^Department of Microbiology and Immunology, Medical University of South Carolina, Charleston, SCUSA; ^2^Hollings Cancer Center, Medical University of South Carolina, Charleston, SCUSA

**Keywords:** ER stress, inflammation, hepatic steatosis, colitis, UPR, inflammasome, IRE1

## Abstract

As an adaptive response to the overloading with misfolded proteins in the endoplasmic reticulum (ER), ER stress plays critical roles in maintaining protein homeostasis in the secretory pathway to avoid damage to the host. Such a conserved mechanism is accomplished through three well-orchestrated pathways known collectively as unfolded protein response (UPR). Persistent and pathological ER stress has been implicated in a variety of diseases in metabolic, inflammatory, and malignant conditions. Furthermore, ER stress is directly linked with inflammation through UPR pathways, which modulate transcriptional programs to induce the expression of inflammatory genes. Importantly, the inflammation induced by ER stress is directly responsible for the pathogenesis of metabolic and inflammatory diseases. In this review, we will discuss the potential signaling pathways connecting ER stress with inflammation. We will also depict the interplay between ER stress and inflammation in the pathogenesis of hepatic steatosis, inflammatory bowel diseases and colitis-associated colon cancer.

## INTRODUCTION

The endoplasmic reticulum (ER) is an intracellular organelle involving in folding of membrane and secreted proteins, synthesis of lipids and sterols, and maintenance of intracellular calcium homeostasis (Kleiner and Brunt, 2012; Milic and Stimac, 2012; Tuyama and Chang, 2012; Wierzbicki and Oben, 2012; Ibrahim et al., 2013). ER chaperones, such as glucose-regulated protein of 78 kDa (GRP78) and 94 kDa (GRP94, also termed as gp96) function as a quality control system that monitors newly synthesized proteins, and ensures only correctly folded proteins to be transported out of ER (Yang and Li, 2005; Yang et al., 2007; Zhang et al., 2011; Cnop et al., 2012; Chen et al., 2013; Lake et al., 2013). ER stress takes place when unfolded or misfolded proteins accumulate in the ER lumen. In response to stress conditions, ER initiates a series of unfolded protein response (UPR) signal transduction pathways, including inositol-requiring enzyme 1 (IRE1), double-stranded RNA-dependent protein kinase (PKR)-like ER kinase (PERK), and activating transcription factor-6 (ATF6) pathways, eventually, leading to changes in translational and transcriptional programs (Urano et al., 2000; McGuckin et al., 2010; Schroder and Sutcliffe, 2010; Hetz, 2012; Zha and Zhou, 2012). Cells that undergo constant ER stress include immune cells such as macrophages, plasma cells, and cells regulating metabolism such as hepatocytes, pancreatic β-cells, adipocytes, and mucosal epithelial cells. Those cells are sensitive to changes in the environments, protein traffic, and ER homeostasis (Ozcan et al., 2006; Eizirik and Cnop, 2010; McAlpine et al., 2010; Schroder and Sutcliffe, 2010; Staron et al., 2010; Ye et al., 2010; Ji et al., 2011; Pfaffenbach and Lee, 2011). While UPR is an adaptive response for cells to restore ER homeostasis, severe or prolonged ER stress leads to cell death and tissue damage. Accumulating evidence indicates that ER stress is involved in various diseases including neurodegenerative diseases, metabolic diseases, inflammatory diseases, cancer, and so on.

Recently, ER stress has been recognized to induce inflammation (McGuckin et al., 2010; Eri et al., 2011; Garg et al., 2012; Osorio et al., 2014; Shenderov et al., 2014). Interestingly, many ER stress-associated diseases also display inflammatory phenotypes (McGuckin et al., 2010; Eri et al., 2011; Garg et al., 2012; Larrain and Rinella, 2012; Petrasek et al., 2012; Szabo and Csak, 2012; Wood, 2012). Inflammatory cytokines released from stressed cells may function as alarming or danger signals to communicate with other cells or to recruit immune cells. While ER stress-induced inflammation is essential for tissue remodeling, it can cause tissue damage and contributes to the pathogenesis of many inflammatory and metabolic diseases (Eizirik and Cnop, 2010; McGuckin et al., 2010; Malhi and Kaufman, 2011; Cnop et al., 2012; Garg et al., 2012; Zha and Zhou, 2012). Inflammation can be induced directly by UPR pathways in stressed cells, or indirectly through interaction with innate immune cells. Induction of innate immunity is mediated by diverse families of Pattern Recognition Receptors (PRRs) that recognize molecular “signature” of the invading pathogens termed as pathogen associated molecular patterns (PAMPs; Takeuchi and Akira, 2010; Kawai and Akira, 2011). Additionally, innate immune cells can be activated by various endogenous ligands from damaged or dead cells. Toll-like receptors (TLRs) are a major family of PRRs mainly expressed by cells of the innate immune system. TLRs can initiate distinct innate immune responses through recruitment of different MyD88 adaptor family members. Currently, at least 13 TLRs have been cloned in mammals, and each receptor is involved in the recognition of a unique set of PAMPs. Studies from our group had demonstrated that GRP94 is a master molecular chaperone in the ER for TLRs. Our results show that the function of most TLRs is dependent on the integrity of GRP94 in the ER (Yang et al., 2007; Liu and Li, 2008). In addition to TLRs, most nucleated cells are capable of sensing and responding to pathogens inside the cytoplasm via intracellular receptors including NOD-like receptors (NLRs) or RIG-I-like receptor family members, which recognize bacteria or viruses, respectively.

TLR activation triggers the recruitment of MyD88 via the Toll-interleukin-1 receptor (TIR) domain, allowing for subsequent recruitment of IL-1R associated kinase (IRAK) and tumor necrosis factor (TNF) receptor associated factor 6 (TRAF6), which leads to the activation of both the NF-κB and JNK pathways. In addition, TLRs are able to activate other signaling cascades including the PI3K, p38, and ERK pathways. Activation of these pathways leads to the expression of inflammatory cytokines such as TNFα and IL-6. Recent progress demonstrates that inflammasomes, signaling complexes essential for IL-1β production, are also involved in various inflammatory and metabolic diseases (Ting et al., 2010; Davis et al., 2011; Stienstra et al., 2011; Henao-Mejia et al., 2012a). Furthermore, pathogens can also induce ER stress directly or indirectly in host cells (Joyce et al., 2009; Merquiol et al., 2011; Muralidharan and Mandrekar, 2013; Blazquez et al., 2014; Chan, 2014; Liu and Dudley, 2014; Smith, 2014). Components of pathogen can interact with TLRs to influence cellular stress response. Martinon et al. (2010) showed that stimulation with TLR4 or TLR2 ligands activated the UPR sensor IRE1α and its downstream target, X box-binding protein (XBP1). The authors indicated that TLR-induced IRE1/XBP1 activation was required for optimal and sustained production of proinflammatory cytokines from macrophages. Notably, XBP1-deficient mice had reduced production of inflammatory mediators and significantly increased bacterial burden when infected with intracellular pathogen *Francisella tularensis*. This novel finding suggests that TLR and IRE1-XBP1 pathways acted in synergy to maximize innate immune responses to pathogens. Interestingly, Woo et al. (2009, 2012) found that the ATF4/CHOP branch of ER stress signaling pathways was selectively suppressed by TLR 3 or 4 through a TRIF-dependent pathway. In mice pretreated with LPS, a TLR4 ligand, ER stress-induced CHOP expression and apoptosis in macrophages, renal tubule cells, and hepatocytes were suppressed. Accordingly, TLR engagement protected mice from ER stress-induced renal dysfunction and hepatosteatosis. Results from those studies suggest that host innate immune pathways modulate ER-stress response to enhance inflammation, thereby the host defense response, while suppress apoptosis pathways during pathogen infections. In addition to the cross-talk of TLR and UPR pathways, viral infection is frequently associated with ER stress and UPR response because of viral protein synthesis and assembly during viral life cycle. A striking example is hepatitis C virus (HCV) infection, which is a strong risk factor for chronic liver diseases and hepatocellular carcinoma. The ER stress and inflammation induced by HCV contribute to chronic liver diseases such as hepatic steatosis and cirrhosis though further studies are needed (Joyce et al., 2009; Merquiol et al., 2011; Chan, 2014).

**FIGURE 1:**
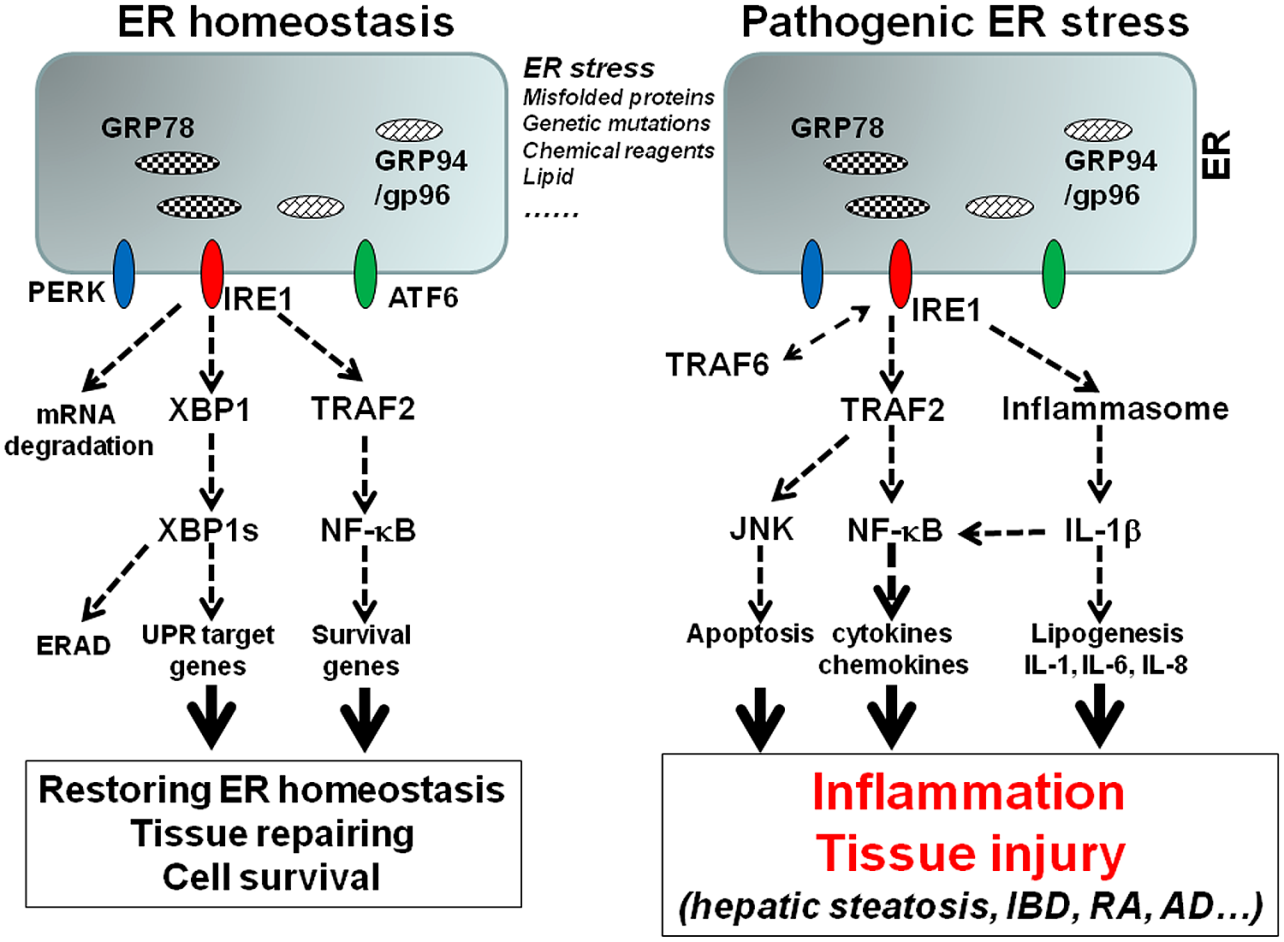
**The model of ER stress-associated inflammation.** Accumulation of misfolded proteins, genetic mutations of ER stress molecules, or pharmacological compounds cause ER stress, which triggers the activation of three major UPR sensors: IRE1α, PERK, and ATF6. Activation of IRE1 pathway leads to the unconventional splicing of XBP-1, which control the transcription of a group of UPR genes including GRP78 and GRP94. The interaction of IRE1 and TRAF2 leads to NF-κB activation, which upregulates survival genes. The signaling and transcription programs initiated by IRE1 and other UPR pathways can restore the ER homeostasis. However, prolonged or unresolved ER stress leads to inflammation. Sustained interaction of IRE1 and TRAF2 leads to NF-κB and JNK activation, which promote inflammatory cytokine production and apoptosis. IRE1 is also involved in ER stress-induced inflammasome activation and IL-1β production, which in turn induces other inflammatory cytokines such as IL-6 and IL-8. In addition, TRAF6 can mediate IRE1 ubiquitination, which is required for TLR mediated optimal inflammatory cytokine production.

Since there are a number of excellent reviewers on ER stress and UPR pathways (Bertolotti et al., 2000; Eizirik and Cnop, 2010; McGuckin et al., 2010; Malhi and Kaufman, 2011; Pfaffenbach and Lee, 2011; Cnop et al., 2012; Garg et al., 2012; Hetz, 2012; Logue et al., 2013; Chan, 2014; Smith, 2014; Zhou and Liu, 2014), this review will focus on ER stress-associated inflammation. ER stress can initiate several signaling pathways that induce inflammation, including ROS production, NF-κB pathway, JNK pathway, and autophagy (McGuckin et al., 2010; Garg et al., 2012; Pagliassotti, 2012). Experimental evidence has indicated that all three major UPR sensors are involved in inflammatory response. However, the IRE1α pathway may play a dominant role in the upregulation of inflammatory cytokines, chemokines, and tissue remodeling molecules. The interaction between IRE1α and TRAF2 activates NF-κB and JNK pathways, which are critical for the expression of cytokines, chemokines, and other inflammatory mediators (Urano et al., 2000; Pincus et al., 2010; Zhang et al., 2011; Lerner et al., 2012; Oikawa et al., 2012; Tam et al., 2012; Upton et al., 2012; Qiu et al., 2013). Our following discussion will be centered on hepatic steatosis and inflammatory bowel diseases to illustrate the interplay between ER stress and inflammation (**Figure 1**).

## ER STRESS-INDUCED INFLAMMATION IN HEPATIC STEATOSIS

As a vital organ for protein synthesis and detoxification, liver is especially susceptible to ER stress. Non-alcoholic fatty liver disease (NAFLD), a spectrum of metabolic disorders ranging from steatosis (NAFL) to steatohepatitis (NASH) to cirrhosis, is the foremost cause of non-alcoholic and non-viral liver-associated illness and death in the US (Kleiner and Brunt, 2012; Larrain and Rinella, 2012; Milic and Stimac, 2012; Pagliassotti, 2012; Tuyama and Chang, 2012; Wierzbicki and Oben, 2012; Ibrahim et al., 2013). Hepatic steatosis or fatty liver can progress to NASH, characterized by progressive liver injury, inflammation, and fibrosis. NASH is also associated with obesity, type 2 diabetes, and liver cancer development (Kleiner and Brunt, 2012; Tuyama and Chang, 2012; Wood, 2012). While ER stress is important for maintaining liver homeostasis, dysregulated ER stress contributes to the pathogenesis of various liver diseases. Accumulating evidence suggests that the interaction between ER stress and inflammation also promote liver steatosis and injury (Malhi and Kaufman, 2011; Cnop et al., 2012; Pagliassotti, 2012; Lake et al., 2013).

### ER STRESS AND HEPATIC STEATOSIS

Although clinical and experimental data implicate the involvement of ER stress in liver diseases, the role and mechanism of ER stress in liver diseases remain not fully understood. A number of studies indicate that ER stress induces liver steatosis through regulating lipid synthesis and inflammation (Malhi and Kaufman, 2011; Li et al., 2012; Pagliassotti, 2012; Wierzbicki and Oben, 2012). This notion is supported by mice carrying genetic mutations of ER stress molecules, or by directly inducing ER stress *in vivo* via administration of chemical reagents (Birkenfeld et al., 2011; Ji et al., 2011; Zhang et al., 2011; Li et al., 2012; Nagarajan et al., 2012; Petrasek et al., 2012; Chen et al., 2013; Guo et al., 2013; Hamano et al., 2013). For example, Tunicamycin (TM), a widely used ER stress inducer, is a nucleoside antibiotics that blocks N-linked glycosylation, causing accumulation of unfolded or misfolded proteins in the ER lumen. Injection of TM into mice results in ER stress-mediated liver steatosis and lipogenesis (Zhang et al., 2011; Lee et al., 2012a, b). These findings indicate the potential pathological role of ER stress in the development of hepatic steatosis. On the other hand, obesity and liver steatosis have been shown to induce ER stress. For instance, mice with high fat feeding not only develop hepatic steatosis, insulin resistance, and type 2 diabetes, but also exhibit ER stress markers in liver and other tissues (Yang et al., 2010; Zhou and Liu, 2010; Birkenfeld et al., 2011). Thus ER stress and hepatic steatosis can form a positive feedback loop to further amplify liver inflammation and injury.

Several genetic strategies have been applied to tease out the roles of ER stress and chaperones in liver steatosis. The ER chaperone protein GRP78 is a critical regulator of ER homeostasis and stress responses, because it interacts and sequesters all major UPR sensors (Ye et al., 2010; Pfaffenbach and Lee, 2011). Kammoun et al. (2009) found that overexpression of GRP78 inhibited ER stress-induced sterol regulatory element binding protein (SREBP) expression and steatosis in the livers of obese (ob/ob) mice. Conversely, Ji et al. (2011) showed that GRP78 deletion led to liver fat accumulation and steatosis. Using conditional GRP78 KO mouse model, Ji et al. (2011) found that liver-specific deletion of GRP78 led to ER stress and apoptosis. These conditional KO mice also displayed liver injury and steatosis. Furthermore, the authors showed that liver-specific deletion of GRP78 exacerbated liver injury and/or steatosis induced by alcohol, high-fat diet, drugs, and toxins (Ji et al., 2011). These findings underscore the critical role of ER tress and GRP78 in liver homeostasis and viability in normal or disease conditions.

Results from Zhang et al. (2011) also demonstrate that ER is not only important for protein quality control, but also critical for lipid synthesis and metabolism. ER stress induces hepatic steatosis through upregulation of transcriptional factors essential for lipogenesis, including CCAAT/enhancer-binding protein β (C/EBPβ), peroxisome proliferator-activated receptor γ (PPARγ), and SREBP. Zhang et al. (2011) showed that the most conserved UPR sensor IRE1α protected animals from ER stress-induced hepatic steatosis. To study the role of IRE1 in liver steatosis, the authors generated a hepatocyte-specific IRE1α deficient mouse line. Deletion of IRE1α gene resulted in profound hepatosteatosis and hypolipidemia in mice under conditions of ER stress induced by proteasome inhibitor Bortezomib, or partial hepatectomy (Zhang et al., 2011). Results from this study further demonstrated that IRE1α represses the expression of transcriptional factors in lipid metabolism pathways, including C/EBPβ, C/EBPδ, and PPARγ (Zhang et al., 2011). The authors proposed that IRE1 is required for maintaining hepatic lipid homeostasis under ER stress conditions.

### THE INTERACTION OF IRE1 AND TRAF PROTEINS IN NF-κB ACTIVATION

IRE1α plays a critical role in transcription of inflammatory genes due to its interaction with TRAF2, which promotes NF-κB activation and inflammatory response. The TRAF family proteins are intracellular adaptors that have been extensively studied in the signaling pathways of TNFR or IL-1/TLR super-families (Chung et al., 2002; Oganesyan et al., 2006; Bishop et al., 2007). All TRAF family proteins (TRAF1-7) have the most conserved TRAF domains in their carboxyl terminal region, which involve in binding to different receptor cytoplasmic tails and the formation of homo- or hetero-dimers between the family members. In addition, all TRAF proteins except TRAF1 have ring fingers, which may function as E3 ubiquitin ligase. Ubiquitination has been shown to play an important role in NF-κB and other signal pathways (Chen, 2005; Pineda et al., 2007; Ha et al., 2009). NF-κB is a homo or heterodimeric transcription factor that binds to κB sites in the promoters of a large number of genes involved in cell survival, inflammation, and immune responses (Senftleben and Karin, 2002; Chen, 2005). The activity of NF-κB is tightly regulated by members of the IκB family. In the classical NF-κB pathway, receptor engagement leads to the activation of the IκB kinase (IKK) complex, which includes IKKα, IKKβ, and IKKγ (NEMO). The activated IKKs phosphorylate IκBs such as IκBα, leading to subsequent ubiquitination and degradation of IκBs. This releases NF-κB and allows it to enter the nucleus and activate transcription of appropriate gene targets (Razani and Cheng, 2010; Dev et al., 2011). NF-κB dimers are composed of the five Rel family members that include NF-κB1 (p50), NF-κB2 (p52), RelA (p65), RelB, and c-Rel. The function of each individual NF-κB molecule is influenced by binding partners and other upstream molecules. Several early studies suggested that UPR sensor IRE1α and signaling molecule TRAF2 are required for activation of NF-κB in response to ER stress-inducing agents, Thapsigargin and TM. Kaneko et al. (2003) showed that ER stress-induced NF-κB activation was inhibited by a dominant-negative form of IRE1 or TRAF2. Hu et al. (2006) also showed that ER stress-induced NF-κB activation was impaired in IRE1α knockdown cells and IRE1 KO MEFs. The authors further demonstrated that TRAF2 provided a critical link between UPR/IRE1 signaling and downstream IKK/NF-κB activation. Biochemical experiments showed that in response to ER stress, IRE1α formed a signaling complex with IKKs though the adapter protein TRAF2 (Hu et al., 2006). Although those studies suggested that the interaction of IRE1, TRAF2, and IKK plays an important role in ER stress-induced NF-κB activation, how IRE1α activates the IKK complex is poorly characterized. Furthermore, the function of IRE1 and TRAF2 in ER stress-induced NF-κB activation *in vivo* needs to be further explored.

### ER STRESS-INDUCED INFLAMMASOME ACTIVATION

IL-1β and other IL-1 family members are major mediators in inflammation. Production of mature IL-1β is regulated by at least two signals: TLR-mediated transcriptional upregulation of pro-IL-1β gene, and inflammasomes-mediated IL-1β maturation (Cassel et al., 2009; Leemans et al., 2011; Rathinam et al., 2012; Wen et al., 2012). An inflammasome is a multiprotein signaling complex, composed of NOD-like protein (NLR) such as NLRP3, the adaptor apoptosis-associated speck-like protein containing a caspase recruitment domain (ASC), and caspase-1. Activation of an inflammasome triggers autoproteolytic cleavage of pro-caspase-1 into its active form, which subsequently processes pro-IL-1β into its mature form (Cassel et al., 2009; Horng and Hotamisligil, 2011; Menu and Vince, 2011; Vandanmagsar et al., 2011). Although very diverse and unrelated stimuli are found to trigger the activation of inflammasomes, the molecular mechanisms responsible for inflammasome activation remain elusive.

Recent progress suggests that inflammasomes play a critical role in a number of autoimmune and metabolic diseases. However, inflammasome activities have been shown to promote or protect liver steatosis (Imaeda et al., 2009; Petrasek et al., 2012; Szabo and Csak, 2012; Wood, 2012). Because of the important role of both ER stress and inflammasome in inflammation, we hypothesize that ER stress and inflammasome activity are interconnected during liver injury and steatosis. While several recent studies demonstrated that ER stress induces pro-IL-1β mRNA expression or NLRP3 inflammasome activation, whether or not UPR pathways are involved in inflammasome activation remains controversial (Lerner et al., 2012; Menu et al., 2012; Oslowski et al., 2012; Shenderov et al., 2014). For instance, an early study from Tschopp’s group suggested that ER stress triggered NLRP3 inflammasome activation in THP-1 cells, a macrophage/monocyte cell line, in a mechanism that is independent of the classical ER stress signaling pathways (Lerner et al., 2012; Menu et al., 2012; Oslowski et al., 2012), whereas other groups suggested that IRE1α under irremediable ER stress induced pro-IL-1β mRNA expression via thioredoxin-interacting protein, which was also associated with programmed cell death of pancreatic β cells. Further studies in our and other laboratories are ongoing to elucidate the molecule mechanisms by which ER stress induces NLRP3 inflammasome activation and IL-1β production.

### URP SIGNALING PATHWAYS INVOLVED IN LIVER INFLAMMATION

In addition to NF-κB pathway, IRE1/TRAF2 can activate other transcription factors such as AP-1, which is also important for the expression of inflammatory cytokines. PERK and ATF6 pathways have also been reported to activate NF-κB (Yamazaki et al., 2009; Nakajima et al., 2011; Tam et al., 2012); however, the mechanisms of PERK or ATF6-medaited NF-κB activation remain unknown. In addition, activation of UPR leads to the generation of ROS, which can induce inflammatory response. All these UPR pathways potentially contribute to liver steatosis and inflammation. However, the relative contribution of each pathway is unclear. Furthermore, NFALD progression is associated with increased apoptosis of hepatocytes. It has been suggested that severe or chronic ER stress can cause cell death via induction of the CHOP pathway (Nishitoh, 2012; Han et al., 2013). The UPR pathways PERK and ATF6 branches are responsible for the activation of CHOP. IRE1 pathway can also induce cell death through either IRE1/TRAF2-mediated JNK activation or direct interaction with Bac and Bak proapoptotic proteins (Kato et al., 2012). It is possible that cell death under severe ER stress conditions leads to sterile inflammation and tissue damage in liver.

## INFLAMMATORY BOWEL DISEASES (IBD)

IBD, including Crohn’s disease (CD) and ulcerative colitis (UC), is a complex intestinal tract disease involving various immune cells, epithelial cells, and intestinal microflora (Strober et al., 2007; Buonocore et al., 2010; Fritz et al., 2011; Papatriantafyllou, 2011; Jostins et al., 2012). Genetic studies have identified many genes, most related with immune response such as IL-10, TLR, NOD2, and IL-23, autophagy and ER stress, that are associated with the development of intestinal inflammation (Hugot et al., 2001; Van Limbergen et al., 2007; Okazaki et al., 2008; Abraham and Cho, 2009; Sarra et al., 2010; Begue et al., 2011; Fritz et al., 2011). Intestinal epithelial cells (IECs) are constantly exposed to gut microflora. One of major functions of intestinal Paneth cells and goblet cells is secretion of various factors essential for the intestinal homeostasis and host defense. Furthermore, macrophages and DCs actively produce inflammatory cytokines in response to translocated bacteria or bacterial components. Therefore, the intestinal system is highly susceptible to ER stress. The interaction of innate and microflora contributes to intestinal inflammation in both acute and chronic colitis. Several excellent studies using genetic mutant mice have highlight the critical role of ER stress in the pathogenesis of colitis.

### GRP94 IN GUT HOMEOSTASIS AND INFLAMMATION

Recent studies from others and our group have demonstrated that ER HSP protein GRP94 is a critical chaperone for multiple TLRs and integrins (Yang et al., 2007). To study the role of ER chaperones in the intestinal homeostasis and inflammation, Liu et al. (2013) generated conditional GRP94 KO mice and intestinal tissue-specific GRP 94 KO mice. In the tamoxifen-inducible GRP94 KO mice, deletion of GRP94 led to rapid weight loss, diarrhea, and ultimately death 12–14 days post-tamoxifen injection. While not affecting embryo development, gut-specific deletion of GRP94 was associated with postnatal death of mutant mice. Pathological analysis demonstrated that GRP94 loss compromised the intestinal barrier function with significant intestinal dilatation, edema and hemorrhage, and thickening of the intestinal wall. The authors further demonstrated that GRP94 maintained gut homeostasis through direct regulation of canonical Wnt-signaling pathway. The results showed that GRP94 interacted with mesoderm development (MesD), an ER chaperone essential for the Wnt coreceptor low-density lipoprotein receptor-related protein 6 (LRP6; Liu et al., 2013). The Wnt/β-catenin signaling pathway has been shown previously to be critical for intestinal homeostasis (Krausova and Korinek, 2014). Mechanistically, GRP94 deletion impaired export of LRP6 from ER to the cell surface, leading to profound loss of gut intrinsic Wnt signaling and intestinal homeostasis. This finding underscores the importance of GRP94 in chaperoning the canonical LRP6/Wnt signal pathway.

### THE IRE1-XBP1 PATHWAY IN INTESTINAL HOMEOSTASIS

Several studies implicated UPR pathways in intestinal inflammation and colitis. Bertolotti et al. (2001) showed that IRE1β deficient mice exhibited increased sensitivity to acute colitis induced by dextran sodium sulfate (DSS). The authors found that expression of IRE1β was restricted to the epithelium of the gastrointestinal tract. DSS treatment led to elevated levels of ER stress markers and increased severity of colitis in IRE1β KO mice, compared with WT mice (Bertolotti et al., 2001). It is unclear whether increased ER stress in IECs was caused by espousing of gut epithelial cells to microflora or by intestinal inflammation after DSS treatment. Nevertheless, these results suggest that deletion of UPR sensor IRE1β disrupted intestinal homeostasis in response to environmental challenge. IRE1 has two forms, IRE1α and IRE1β, encoded by two distinct genes. It is noteworthy that untreated IRE1β deficient mice showed no sign of intestinal inflammation histologically (Bertolotti et al., 2001). One of reasons might be the redundant role of IRE1α and IRE1β. However, whether specific deletion of IRE1α in the IECs enhances the susceptibility to DSS-induced colitis has not been reported.

The involvement of IRE1 pathway in colitis is also supported by XBP1 conditional KO mice (Kaser et al., 2008). As a ribonuclease, an important substrate of IRE1 is the mRNA for transcriptional factor XBP1. Activated IRE1 splices the XBP1 mRNA by the excision of a 26bp fragment via an unconventional splicing mechanism, generating spliced XBP1 (XBP1s; Adolph et al., 2012; Upton et al., 2012). XBP1s controls the transcription of a set of UPR target genes, including chaperones, protein disulfide isomerases (PDIs), and components of ERAD, essential for the maintenance of ER function. Furthermore, XBP1 plays a critical role in the development of highly secretory cells such as plasma cells and pancreatic cells (Glimcher, 2010; Sha et al., 2011). Dr. Blumberg’s group reported that mice with tissue-specific deletion of XBP1 in IECs developed spontaneous enteritis and displayed increased susceptibility to DSS-induced colitis (Kaser et al., 2008). Kaser et al. (2008) showed that mice deficient in XBP-1 in intestinal tissues displayed spontaneous intestinal inflammation characterized by immune cell infiltration, loss of crypts, and ulceration. Strikingly, deletion of XBP1 in IECs also resulted in the reduced number of Paneth and goblet cells (Kaser et al., 2008; Adolph et al., 2013). A major function of Paneth cells is to secret antimicrobial peptides. Goblet cells are IECs that produce protective mucus in the microbiota–intestine interface. The diminished function or number of both cell types rendered these XBP1 deficient mice more susceptible to DSS-induced colitis. The authors also found that XBP-1 deficient intestinal tissues had increased expression of ER stress mediators such as GRP78, ATF4, and CHOP (Kaser et al., 2008). Enhanced ER stress may lead to more inflammation in response to gut microflora. In addition, upregulation of CHOP may contribute to apoptosis of Paneth cells in XBP-1 deficient mice. Thus, dysregulated ER stress in IEC compartment induces intestinal inflammation and colitis. Kaser et al. (2008) also found the association of XBP1 variants with both CD and UC, indicating XBP1 as a genetic risk factor for human IBD.

### INFLAMMASOME ACTIVATION IN THE DEVELOPMENT OF COLITIS

The role of inflammasomes as well as their interaction with microbiota in intestinal inflammation is an active area of research. Up to now, a number of publications have painted a more complex picture of inflammasomes in colitis and colitis-associated colon cancer (Sands, 2007; Bauer et al., 2010, 2012; Zaki et al., 2010; Henao-Mejia et al., 2012b). Moreover, the interaction between ER stress pathways and inflammasomes is still yet to be established. Several studies showed that mice deficient for inflammasome components including NLRP3, ASC, and caspase-1 were highly susceptible to acute colitis induced by DSS, indicating the protective role of inflammasome in acute colitis (Allen et al., 2010; Dupaul-Chicoine et al., 2010; Hirota et al., 2011). However, Bauer et al. (2010) showed that that defective in NLRP3 inflammasome protected mice from DSS-induced acute colitis, indicating that inflammasomes contribute to the development of colitis. Our recent study showed that inflammasome activation promoted the intestinal inflammation in IL-10 KO mice, which develops chronic colitis resembling human IBD. We also found that inhibition of inflammasome activities with IL-1 receptor antagonist or caspase-1 inhibitors suppressed intestinal inflammation (Zhang et al., 2014). Our data further suggest that inflammasome-derived IL-1β promoted Th17 phenotype in intestinal tissues. Those results indicate that without inhibitory effects of IL-10, the interaction between inflammasomes and microbiota leads to intestinal inflammation and the development of colitis.

## ER STRESS AND INFLAMMATION IN COLITIS-ASSOCIATED COLON CANCER

Inflammation contributes to tumor initiation and progression, especially for colitis-associated colon cancer. Because of unregulated growth and hypoxia environments, ER stress is associated with tumor development. Depending on type or stage of tumor development, ER stress can enhance or suppress tumor development (Ghiringhelli et al., 2009; Allen et al., 2010; Hu et al., 2011; Bruchard et al., 2013). Moreover, Mahadevan et al. (2011) found that ER stress from stressed tumor cells could be transmitted to other neighborhood cells via un-identified heat-stable molecules. Conditioned media derived from cancer cells with ER stress could induce ER stress markers such as XBP-1, GRP78, and CHOP in macrophages, leading to production of inflammatory molecules (Mahadevan et al., 2011). It is possible that this kind of transmissible ER stress could amplify inflammation in tumor microenvironments.

Because most studies on ER stress and tumor development are focused on tumor-intrinsic stress pathways, research on ER stress and inflammation in tumor microenvironments is limited so far. Using macrophage-specific KO mice, Morales et al. (2014) showed that mice with macrophage-specific deletion of GRP94/gp96 exhibited decreased colitis and inflammation-associated colon cancer induced by DSS/AOM, with reduced expression of inflammatory cytokines such as IL-6, IL-17, and IL-23. These results demonstrate the macrophage-intrinsic role of chaperone GRP96 in promoting colitis and inflammation-associated colon tumorigenesis.

Similar to its controversial role in colitis, inflammasomes also have a complex role in tumor development. A study by Allen et al. (2010) showed that NLRP3-deficient mice were more sensitive to colorectal tumorigenesis. The authors found that mice deficient for NLRP3 inflammasome components including NLRP3, ASC or caspase-1 had severe colitis and increased tumorigenesis in AOM/DSS colon cancer model. But NLRC4 KO mice displayed similar incidence of colitis-associated colon cancer as WT mice. The authors further demonstrated that NLRP3-inflammasome activation in bone marrow-derived cells was critical for the tumor suppression. In contrast, Hu et al. (2011) found that caspase-1 KO and NLRC4 KO mice, but not NLRP3 KO mice, had increased tumorigenesis in the AOM/DSS colon cancer model. Furthermore, NLR6 and NLR12 also have been shown to suppress tumor development in AOM/DSS colon cancer model (Chen and Nunez, 2011; Chen et al., 2011). A possible explanation is that inflammasome-processed IL-1 and IL-18 signaling in IECs provides protection against apoptosis of gut epithelial cells. Those studies imply that the inflammasomes may promote or suppress colon cancer development depending on experimental conditions, possibly gut microflora.

## ER STRESS AND INFLAMMATION IN OTHER DISEASES

ER stress-induced inflammation is also associated with other metabolic (Kawasaki et al., 2012; Papa, 2012; Tripathi and Pandey, 2012; McAlpine and Werstuck, 2013; Zhou and Tabas, 2013; Biden et al., 2014), inflammatory (Blohmke et al., 2012; Blumental-Perry, 2012; Yoo et al., 2012; Qiu et al., 2013; Savic et al., 2014), neurodegenerative diseases (Bellucci et al., 2011; Cunnea et al., 2011; McMahon et al., 2012; Vidal et al., 2012; Cornejo and Hetz, 2013; Mercado et al., 2013; Li et al., 2014), pathogen infections (Martinon et al., 2010; Merquiol et al., 2011; Tesh, 2012; Lee et al., 2013; Blazquez et al., 2014; Chan, 2014), and cancer (Shuda et al., 2003; Kubisch and Logsdon, 2008; Romero-Ramirez et al., 2009; Hardy et al., 2012; Tang et al., 2012; Chen et al., 2013; Mahoney et al., 2013; Kharabi Masouleh et al., 2014; Martin-Perez et al., 2014; Shimodaira et al., 2014; Zheng et al., 2014). While this review focuses on liver steatosis and inflammatory bowl diseases, **Table 1** listed various diseases where pathogenesis has directly or indirectly been linked to ER stress.

**Table 1:** A list of diseases linked to dysregulated ER stress.

Diseases	Reference
**Metabolic diseases**
Hepatic steatosis	Malhi and Kaufman (2011), Zhang et al. (2011)
Type 2 diabetes mellitus	Papa (2012), Biden et al. (2014)
Obesity	Kawasaki et al. (2012), Tripathi and Pandey (2012)
Atherosclerosis	McAlpine and Werstuck (2013), Zhou and Tabas (2013)
**Autoimmune diseases**
Arthritis	Yoo et al. (2012), Qiu et al. (2013), Savic et al. (2014)
Inflammatory diseases	Kaser et al. (2008), Liu et al. (2013)
Chronic pulmonary diseases	Blohmke et al. (2012), Blumental-Perry (2012)
**Neurodegenerative diseases**
Alzheimer’s disease	Cornejo and Hetz (2013), Li et al. (2014)
Parkinson’s disease	Bellucci et al. (2011), Vidal et al. (2012), Mercado et al. (2013)
Multiple sclerosis	Cunnea et al. (2011), McMahon et al. (2012)
**Pathogen infections**
Viral infection	Chan (2014), Tesh (2012), Blazquez et al. (2014)
Bacterial infection	Martinon et al. (2010), Lee et al. (2013)
**Tumor**
Breast cancer	Martin-Perez et al. (2014), Zheng et al. (2014)
Colon cancer	Hardy et al. (2012), Morales et al. (2014)
Leukemia and lymphomas	Mahoney et al. (2013), Kharabi Masouleh et al. (2014)
Hepatocellular carcinoma	Shuda et al. (2003), Tang et al. (2012), Chen et al. (2013)
Pancreatic cancer	Kubisch and Logsdon (2008), Romero-Ramirez et al. (2009)

## CONCLUSION AND PERSPECTIVES

ER stress functions as an adaptive response to maintain cell homeostasis and survival when normal protein or lipid synthesis and metabolism are perturbed. Emerging evidence indicates that ER stress is also closely associated with inflammation, in addition to its classic role in protein quality control. We propose that the IRE1/TRAF2 axis represents a major pathway to link ER stress with key transcription factors such as NF-κB that controls the expression of inflammatory cytokine genes. We also speculate that the interaction between UPR pathways and inflammasomes provides another link between ER stress and inflammation. While the activation of UPR pathways can lead to resolving the ER stress and achieving new protein homeostasis, prolonged and severe ER stress can result in cell death and tissue damage, contributing to the pathogenesis of various diseases. In both scenarios, inflammation can be induced in stressed cells and surrounding cells. In a broad term, inflammatory diseases such as IBD and hepatic steatosis are the results of interaction amongst ER stress, inflammatory cytokines, metabolism, and gut microflora (Machado and Cortez-Pinto, 2012). Similarly, ER stress and inflammation can promote or inhibit the development of colitis-associated cancer depending on genetic background, environments, and intestinal microflora. A better understanding of ER stress and inflammation may lead to identification of potential therapeutic targets for the treatment of inflammatory and metabolic diseases as well as cancer.

## Conflict of Interest Statement

The authors declare that the research was conducted in the absence of any commercial or financial relationships that could be construed as a potential conflict of interest.
